# Maternal dietary patterns and acute leukemia in infants: results from a case control study in Mexico

**DOI:** 10.3389/fnut.2023.1278255

**Published:** 2023-11-13

**Authors:** Paloma Muñoz-Aguirre, Edgar Denova-Gutiérrez, María Luisa Pérez-Saldivar, Laura E. Espinoza-Hernández, Elisa M. Dorantes-Acosta, José R. Torres-Nava, Karina A. Solís-Labastida, Rogelio Paredes-Aguilera, Martha M. Velázquez-Aviña, R. Martha Espinosa-Elizondo, M. Raquel Miranda-Madrazo, Ana Itamar González-Ávila, Luis Rodolfo Rodríguez-Villalobos, Juan José Dosta-Herrera, Javier A. Mondragón-García, Alejandro Castañeda-Echevarría, M. Guadalupe López-Caballero, Sofía I. Martínez-Silva, Juan Rivera-González, Norma Angélica Hernández-Pineda, Jesús Flores-Botello, Jessica Arleet Pérez-Gómez, María Adriana Rodríguez-Vázquez, Delfino Torres-Valle, Jaime Ángel Olvera-Durán, Annel Martínez-Ríos, Luis R. García-Cortés, Carolina Almeida-Hernández, Janet Flores-Lujano, Juan Carlos Núñez-Enriquez, Minerva Mata-Rocha, Haydeé Rosas-Vargas, David Aldebarán Duarte-Rodríguez, Silvia Jiménez-Morales, Juan Manuel Mejía-Aranguré, Lizbeth López-Carrillo

**Affiliations:** ^1^Consejo Nacional de Humanidades, Ciencias y Tecnologías (CONAHCYT), Mexico City, Mexico; ^2^Centro de Investigación en Salud Poblacional, Instituto Nacional de Salud Pública, Cuernavaca, Mexico; ^3^Centro de Investigación en Nutrición y Salud, Instituto Nacional de Salud Pública, Cuernavaca, Mexico; ^4^Unidad de Investigación Médica en Epidemiología Clínica, Hospital de Pediatría, Centro Médico Nacional (CMN) Siglo-XXI, Instituto Mexicano del Seguro Social (IMSS), Mexico City, Mexico; ^5^Servicio de Hematología Pediátrica, Hospital General “Gaudencio González Garza”, CMN “La Raza”, IMSS, Mexico City, Mexico; ^6^Departamento de Hemato-Oncología, Hospital Infantil de México Federico Gómez, Secretaria de Salud (SSA), Mexico City, Mexico; ^7^Servicio de Oncología, Hospital Pediátrico Moctezuma, Secretaría de Salud de la Ciudad de México (SSCDMX), Mexico City, Mexico; ^8^Servicio de Hematología, UMAE Hospital de Pediatría, CMN “Siglo XXI”, IMSS, Mexico City, Mexico; ^9^Servicio de Hematología, Instituto Nacional de Pediatría (INP), SSA, Mexico City, Mexico; ^10^Servicio de Onco-Pediatría, Hospital Juárez de México, SSA, Mexico City, Mexico; ^11^Servicio de Hematología Pediátrica, Hospital General de México, SSA, Mexico City, Mexico; ^12^Servicio de Hematología Pediátrica, CMN “20 de noviembre”, Instituto de Seguridad Social al Servicio de los Trabajadores del Estado (ISSSTE), Mexico City, Mexico; ^13^Servicio de Hematología Pediátrica, HGR No. 1 “Dr. Carlos Mac Gregor Sánchez Navarro” IMSS, Mexico City, Mexico; ^14^Servicio de Pediatría, Hospital Pediátrico de Tacubaya, SSCDMX, Mexico City, Mexico; ^15^Servicio de Cirugía Pediátrica, Hospital General “Gaudencio González Garza”, CMN “La Raza”, IMSS, Mexico City, Mexico; ^16^Servicio de Cirugía Pediátrica, Hospital General Regional (HGR) No. 1 “Dr. Carlos Mac Gregor Sánchez Navarro” IMSS, Mexico City, Mexico; ^17^Servicio de Pediatría, Hospital General de Zona Regional (HGZR) No. 25 IMSS, Mexico City, Mexico; ^18^Coordinación Clínica y Pediatría, Hospital Pediátrico de Coyoacán, SSCDMX, Mexico City, Mexico; ^19^Hospital Pediátrico de Iztapalapa, SSCDMX, Mexico City, Mexico; ^20^Hospital General “Dr. Gustavo Baz Prada”, Instituto de Salud del Estado de México (ISEM), Estado de México, Mexico; ^21^Coordinación Clínica y Pediatría del Hospital General de Zona 76 IMSS, Mexico City, Mexico; ^22^Coordinación Clínica y Pediatría, Hospital General “La Perla” ISEM, Estado de México, Mexico; ^23^Coordinación Clínica y Pediatría, HGR No. 72 “Dr. Vicente Santos Guajardo”, IMSS, Mexico City, Mexico; ^24^Coordinación Clínica y Pediatría del Hospital General de Zona 68, IMSS, Mexico City, Mexico; ^25^Coordinación Clínica y Pediatría del Hospital General de Zona 71, IMSS, Mexico City, Mexico; ^26^Servicio de Cirugía Pediátrica, HGR 1° Octubre, ISSSTE, Mexico City, Mexico; ^27^Hospital Regional “General Ignacio Zaragoza”, ISSSTE, Mexico City, Mexico; ^28^Delegación Regional Estado de México Oriente, IMSS, Estado de México, Mexico; ^29^Hospital General de Ecatepec “Las Américas”, ISEM, Estado de México, Mexico; ^30^Laboratorio de Biología Molecular de las Leucemias, Unidad de Investigación en Genética Humana, UMAE, Hospital de Pediatría, CMN “Siglo XXI”, IMSS, Mexico City, Mexico; ^31^Laboratorio de Genética, Hospital de Pediatría, Centro Médico Nacional (CMN) Siglo-XXI, Instituto Mexicano del Seguro Social (IMSS), Mexico City, Mexico; ^32^Laboratorio de Innovación y Medicina de Precisión, Núcleo A, Instituto Nacional de Medicina Genómica (INMEGEN), Mexico City, Mexico; ^33^Facultad de Medicina, Universidad Nacional Autónoma de México (UNAM), Mexico City, Mexico

**Keywords:** dietary patterns, pediatric cancer, acute leukemia, leukemia, maternal diet, case–control

## Abstract

**Background:**

Childhood cancer is the leading cause of disease-related mortality among children aged 5–14 years in Mexico, with acute leukemia being the most common cancer among infants. Examining the overall dietary patterns allows for a comprehensive assessment of food and nutrient consumption, providing a more predictive measure of disease risk than individual foods or nutrients. This study aims to evaluate the association between maternal dietary patterns during pregnancy and the risk of acute leukemia in Mexican infants.

**Methods:**

A hospital-based case–control study was conducted, comparing 109 confirmed acute leukemia cases with 152 age-matched controls. All participants (≤24 months) were identified at hospitals in Mexico City between 2010 and 2019. Data on *a posteriori* dietary patterns and other relevant variables were collected through structured interviews and dietary questionnaires. Multivariate logistic regression was employed to estimate the association between maternal dietary patterns during pregnancy and the risk of acute leukemia in infants.

**Results:**

The “Balanced & Vegetable-Rich” pattern, characterized by a balanced consumption of various food groups and higher vegetable intake, exhibited a negative association with acute leukemia when compared to the “High Dairy & Cereals” Pattern (adjusted odds ratio [OR] = 0.51; 95% confidence interval [CI]: 0.29, 0.90). We observed that mothers who gave birth to girls and adhered to a healthy dietary pattern during pregnancy exhibited significantly lower odds of their children developing AL compared to those who gave birth to boys [OR = 0.32 (95% CI 0.11, 0.97)]. Our results underscore the significance of maternal nutrition as a modifiable factor in disease prevention and the importance of prenatal health education.

## 1. Introduction

In Mexico, childhood cancer is the first cause of death by disease in children aged 5–14 years and the sixth among those under five. It represents almost 70% of the total burden of disease in these age groups (1). Acute Leukemia (AL) is the most common cancer among children and adolescents around the world. Among Hispanic children, the incidence of AL is the highest compared with other neoplasms in Caucasian and African American and is more frequent in males than among females (2). In spite that this disease represents a low proportion of cancer cases in the total population, it causes the highest number of years of life potentially lost (YLL) due to premature death.

AL etiology is largely unknown. However, experimental evidence show that translocations are initiating events that occur early *in utero* and are present in a substantial proportion of childhood AL. Clonal markers in leukemic cells and clonotypic fusion gene sequences in neonatal blood spots, have been observed in monozygotic twins, in whom only one of them develops the disease (3). These findings underscore the need to examine potential risk factors in the fetal environment, such as maternal diet. Diet may yield protective and/or negative effects leading to cancer, not only through the intake of diverse dietary components that take part in epigenetic processes such as: DNA methylation, histone modifications, noncoding RNAs in fetus but also, as a vehicle for carcinogenic compounds, like N-nitroso precursors, that cross transplacental barrier (4).

Evidence suggests that the size and growth rate of the fetus during gestation may play a role in the development of leukemia. For example, some studies have shown that children who are born small for gestational age may have a higher risk of developing leukemia (5). It is thought that a slower growth rate during gestation may increase the risk of certain genetic mutations that can lead to the development of leukemia. Some studies have reported that maternal nutrition effects on fetal growth and development may differ between male and female fetuses (6). However, there is limited evidence on the differential effects of maternal dietary patterns on leukemia risk in boys and girls.

Early epidemiological studies focused on AL and maternal diet evaluation of a single nutrient and/or food. Further studies evidenced the role of food groups on AL development. In this way, there is available evidence regarding the negative association between maternal consumption of folic acid and protein with AL, respectively (7), as well as the consumption of fruits, vegetables, fish, shellfish, beans, and beef (8). In contrast to the observed relationship with the consumption of chocolate, wine, coffee, and processed meats, which are dietary inhibitors of the nuclear enzyme topoisomerase II, as well as sugars and syrups (9). Individual components of the diet interact with each other, so this approach of evaluating dietary components one by one has gradually evolved to the evaluation of dietary patterns.

Because of the complexity of diets, the overall effects (antagonic or synergistic) of various nutrients and foods that are consumed simultaneously, may only be assessed through the identification of dietary patterns (10), which depend on culture and availability of foods in each population and are not necessarily replicated throughout different countries. Research on dietary patterns in Latin American populations is scarce, and its possible relationship with childhood AL is null.

Our aim was to evaluate the association between maternal dietary patterns during pregnancy and AL in Mexican infants and to explore if this association differs by sex.

## 2. Materials and methods

During the period of 2010 to 2019, a clinical based case–control study was carried out in Mexico City and State of Mexico. The study population comprised children that were identified in 9 public secondary and tertiary public hospitals. The protocol was approved by the Mexican Institute of Social Security (IMSS) IRB with number 2010-785-064.

### 2.1. Cases

Eligible cases were children up to 24 months of age with acute leukemia (AL), which was confirmed with bone marrow smears and histochemical tests (myeloperoxidase, sudan black B reaction, esterases, periodic Schiff reaction (PAS) and acid phosphatase) and immunophenotype. In total, 237 eligible cases were identified and 110 accepted to participate, yielding a response rate of 47.8%. Not participating children included: 11 which denied participation and 116 did not have complete dietary information.

### 2.2. Controls

Cases were sex and 1:1 age matched (±12 months) with a child (control) attending to any of the ambulatory surgery services from the same health institution where the cases were gathered (IMSS, Secretary of Health, Secretary of Health of Mexico City, State of Mexico Institute of Health, Institute of Security and Social Services for State Workers). In total, 276 eligible controls were identified, of which 24 did not agree to participate (response rate of 91.3%) leaving 252 controls whose diagnoses were: circumcision (%), hernioplasty, orchiopexy, tonsillectomy, intoxication, first and second level burns.

### 2.3. Interviews

Mothers gave informed consent to participate in a face-to-face interview in the hospitals. Previously trained personnel gathered information regarding the family sociodemographic characteristics, child reproductive history, parents’ alcohol, and tobacco consumption as well as maternal diet during pregnancy.

### 2.4. Maternal diet

Dietary maternal intake, during pregnancy, was obtained through a food frequency questionnaire (FFQ). This instrument contained 116 items including foods, beverages, and local dishes. The reproducibility of this questionnaire was previously evaluated in Mexican women, to whom it was applied twice at an interval of 1 year, while its validity was estimated using a 24-h diet recall at 3-month intervals as a reference. The details of this validation have been published (11).

According to the methodology suggested by Willett et al. (12) the FFQ includes 10 response options for frequency of consumption ranging from “never” to “6 or more times a day,” as well as predetermined portions for each food as follows: a glass (e.g., milk and wine), cup (for yogurt, some fruits and vegetables, tea, juices, alcoholic and non-alcoholic beverages), a spoon (e.g., oils, sour cream, sauces and nuts), a slice (e.g., cheese, some fruits and meats), a plate (e.g., legumes and local dishes) and a piece (e.g., some fruits and breads).

Total energy content in foods and local dishes was obtained from nutritional composition tables of the United States Department of Agriculture (USDA 2007, 2017–2018) that include a wide variety of foods similar to those consumed in the study area. For the few foods that were not found in the USDA tables, such as tejocote (a local fruit), we used the reference tables of the National Institute of Medical Sciences and Nutrition “Salvador Zubirán” (13). Only two foods (soy juice and soy beer) were not found in neither food data sources.

Energy consumption was estimated by adding the caloric intake from foods and local dishes. Due to the fact, that some fruits and vegetables are only consumed during certain seasons of the year, their energy intake was weighted according to their availability in the market, for example, only 50% of the kcal of plums were considered, as they are available for 6 months of the year (14).

Up to this phase, one case was eliminated, because the estimated total energy intake was less than 525 kcal, which corresponds to less than 2 standard deviations of the daily intake observed in pregnant Mexican women and may not represent a valid biological value (15). Therefore, the final sample size of this report was 109 cases and 252 controls.

#### 2.4.1. Dietary patterns

We derived dietary patterns from 22 food groups and 8 isolated foods. Food groups were created according to their similarity in the content of macro and micronutrients (e.g., fat, carbohydrates, protein, vitamins, sodium); sugar (added or not) or type of fat (saturated or vegetal); otherwise, foods were included as isolated items (atole, corn, corn tortilla, egg, poultry, avocado, dehydrated cranberries and soy sauce).

To derive dietary patterns we used 2 different approaches: Cluster analysis and factor analysis. For cluster analysis, we used food groups and foods in portions per day and their energy percentage contribution. Through the K-means method, we ran 6 cluster solutions and selected one that contained 2 non-overlapping clusters. As a result, each individual belonged to only one cluster that we named Balanced & Vegetable-Rich or High Dairy & Cereals.

We further determined the factor loadings of each food group, using factor analysis (14). We orthogonally rotated the factors (varimax rotation) to keep them uncorrelated and to improve their interpretation. After assessment of graphic analysis and interpretability, we retained factors with eigenvalues >1.5. We defined each factor by a subset of at least 5 food groups with an absolute loading ≥0.2 (16).

We estimated each pattern by summing the personal intake of the food groups weighted by their corresponding loading factor. We derived 3 dietary patterns named High Saturated Fats & Sugars, Moderate Meat & Cereals and Balanced & Vegetable-Rich. With this analysis, each participant receives a score for each pattern identified.

### 2.5. Statistical analysis

Mother, father, and child characteristics were compared between cases and controls using Chi square, one-way ANOVA (Table 1).

**Table 1:** General characteristics of the study subjects.

Characteristics	Cases (*n* = 109)		Controls (*n* = 252)		*p*-value
	(*n*)		(*n*)		
*Child*					
Sex, boys %	52	47.7	151	60.4	0.026
Age, months [mean ± SD]	109	15.5 ± 7.3	252	18.5 ± 9.7	0.004
State of residence, %					
Mexico City	42	38.5	132	52.4	0.016
State of Mexico	67	61.5	120	47.6	
Health institution*, %					
Ministry of Health	62	56.9	164	65.08	0.139
Mexican Institute of Social Security	47	43.1	88	34.9	
Person/room ratio, [p50(p10,p90)]	109	2.5 (1.5,5.0)	252	3.00 (1.7,6.0)	0.047
Breastfeeding, yes %	96	88.1	226	89.7	0.651
Breastfeeding, months [p50(p10,p90)]	109	6.0 (0.0,15.0)	252	7.0 (0.0,18.0)	0.242
Birth weight, grams [mean ± SD]	109	3,144.8 ± 400.7	252	2,928.1 ± 610.9	0.001
*Mother*					
Age at pregnancy, years [mean ± SD]	109	25.9 ± 6.4	252	25.6 ± 6.3	0.615
Education, years [p50 (p10,p90)]	109	9.00 (6.0,15.0)	252	10.00 (7.0,13.6)	0.833
Smoking before pregnancy, yes %	31	28.4	77	30.6	0.687
Smoking during pregnancy, yes %	4	3.7	7	2.8	0.74
Alcohol consumption during pregnancy, yes %	4	3.7	31	12.3	0.011
Iron supplement during pregnancy, yes %	76	69.7	216	85.7	<0.001
Mineral consumption during pregnancy, yes %	4	3.67	16	6.4	0.453
Vitamins supplement during pregnancy, yes %	98	89.9	238	94.4	0.119
Drug use for genital infection, yes %	15	13.8	34	13.5	0.945
*Father*					
Age at pregnancy, years [mean ± SD]	105	28.8 ± 7.9	250	28.8 ± 7.4	0.944
Education, years [p50 (p10,p90)]	104	10.5 (6.0,15.0)	245	9.0 (6.0,14.0)	0.226
Smoking before pregnancy, yes %	48	47.1	141	58	0.062
Alcohol consumption before pregnancy, yes %	88	85.4	222	90.6	0.158

*Includes Secretary of Health, Secretary of Health of Mexico City, State of Mexico Institute of Health (ISEM, by its acronym in Spanish), Institute of Security and Social Services for State Workers (ISSSTE, by its acronym in Spanish).

The association between maternal dietary patterns and AL was assessed using unconditional multivariate logistic regression models. Covariates were selected based on two criteria. Firstly, we included covariates that exhibited significant differences between the cases and controls. These covariates encompassed factors such as institution, breastfeeding, age at pregnancy, education (both maternal and paternal), smoking before and during pregnancy, iron and vitamin consumption, drug use for genital infection, and the age of the father. Secondly, we incorporated covariates based on a causal directed acyclic graph (DAG) approach, which considered variables such as age at pregnancy, state of residence, person/room ratio, breastfeeding, iron and vitamin supplementation, tobacco and alcohol use, and maternal education. Detailed information regarding the covariates can be found in Supplementary Figure 1.

Using as a reference category the “High Dairy & Cereals” cluster, we estimated the odds ratio for AL among individuals belonging to the “Balanced & Vegetable-Rich” cluster. We created tertiles based on the dietary pattern score distributions among controls. We estimated the odds ratios for LA comparing tertile 3 and 2 versus tertile 1, respectively. We also stratified the adjusted models and estimated the dietary pattern x sex interaction adding the respective multiplicative term.

We performed tests for linear trends using the continuous dietary pattern scores. We used *p* < 0.05 as a cutoff for significance.

We conducted our analysis using STATA, version 13 and Daggity v3.0.

## 3. Results

In order to ensure comparability between the study groups, we carefully controlled for the distribution of children’s age and sex, which was similar across all groups. Comparative analysis revealed that mothers of children with AL exhibited lower alcohol intake during pregnancy, lower person/room ratios and lower intakes of iron during pregnancy compared to mothers of healthy children. Moreover, children diagnosed with AL were reported to have a higher birthweight compared to controls (see Table 1).

Through cluster analysis, we identified two distinct dietary patterns, labeled as ‘High Dairy & Cereals’ and ‘Balanced & Vegetable-Rich.’ Both patterns included food groups such as ‘other fruits’ and ‘other vegetables.’ However, the ‘High Dairy & Cereals’ cluster stood out for its higher consumption of cereals, dairy products, and eggs, whereas the ‘Balanced & Vegetable-Rich’ cluster was characterized by a higher intake of allium vegetables and corn tortillas.

Additionally, factor analysis yielded three major dietary patterns: (1) ‘Balanced & Vegetable-Rich,’ which was characterized by high consumption of fruits, allium vegetables, other vegetables, and legumes, and low consumption of pastries and refined cereals; (2) ‘High Saturated Fats & Sugars,’ which exhibited high consumption of saturated fats, refined cereals, canned products, corn tortillas, and sodas, and low consumption of whole grain cereals, seafood, and dairy products; and (3) ‘Moderate Meat & Cereals,’ which showed high consumption of processed meat, red meat, poultry, and refined cereals, and low consumption of dairy, fruits, and legumes. The factor-loading matrixes for these dietary patterns explained a total variance of 20.3% (see Table 2).

**Table 2:** Food groups consumption (portions/day) according to dietary patterns using cluster and factor analysis in the study sample (cases = 109, controls = 252).

	Cluster		Dietary pattern						
Food groups	High dairy & cereals	Balanced & vegetable-rich	High saturated fat & sugars		Moderate meat & cereals		Balanced & vegetable-rich		All women
	Portions/day (mean)		Factor loadings/portions/day (mean)						Portions/day (mean)
Dairy products	1.1	1.16		1.18	0.49	1.13		1.1	1.14
Dairy with added sugar	0.43	0.47	0.33	0.39	0.55	0.4		0.41	0.46
Citrus fruits	0.81	1.03		0.9	0.34	0.9	0.41	0.97	0.96
Other fruits	1.95	2.09		1.88	0.5	1.76	0.49	1.85	2.04
Egg	0.78	0.7	0.32	0.69		0.68		0.84	0.73
Poultry	0.37	0.41		0.4	0.35	0.39		0.4	0.39
Processed meats	0.61	0.59	0.22	0.55	0.61	0.5	0.21	0.6	0.6
Red meat	0.53	0.46		0.43	0.5	0.43		0.52	0.48
Fish and shellfish	0.25	0.27	−0.21	0.23	0.51	0.21		0.26	0.26
Saturated fats	0.71	0.74		0.69	0.63	0.66		0.74	0.72
Cruciferous vegetables	0.43	0.35		0.39		0.34	0.65	0.34	0.37
Allium vegetables	1.74	1.39	0.74	1.52		1.3		1.45	1.49
Green leafy vegetables	0.6	0.67		0.62		0.56	0.73	0.51	0.64
Other vegetables	3.48	2.5	0.7	2.5		2.45	0.38	2.83	2.79
Corn	0.18	0.16		0.16		0.16	0.51	0.16	0.17
Potato	0.33	0.29	0.38	0.3		0.29	0.54	0.31	0.3
Legumes	0.81	0.73	0.34	0.73	0.21	0.69	0.51	0.71	0.75
Canned chili peppers	0.28	0.17	0.74	0.14		0.14		0.18	0.2
Corn tortilla	12.62	3.24	0.36	5.12		5.48		5.74	6.05
Cereals	2.03	2.02		2.04	0.52	1.91	0.22	1.85	2.02
Cereals high in fat and sugar	0.74	0.61	0.48	0.62	0.44	0.61		0.58	0.65
Soft drinks	0.56	0.34	0.61	0.31		0.36		0.32	0.41
Coffee and tea	0.91	0.72	0.43	0.71		0.68	−0.24	0.75	0.77
Corn-based drinks	0.17	0.22		0.16	0.29	0.17	0.37	0.19	0.21
Vegetable oils	0.88	0.64	0.62	0.6		0.61		0.7	0.71

Our analysis unveiled a notable inverse association between the ‘Balanced & Vegetable-Rich’ dietary pattern and the risk of developing Acute Leukemia (AL). While adjusting for various influencing factors, including institution, breastfeeding, age at pregnancy, parental education, smoking habits before and during pregnancy, iron and vitamin consumption, drug use for genital infections, age of the father, and paternal alcohol consumption, the ‘Balanced & Vegetable-Rich’ pattern demonstrated odds ratios (OR) of 0.51 (95% CI, 0.29, 0.90) in the cluster analysis. In the factor analysis, assessing different levels of adherence (T3 vs. T1) to the same dietary pattern (‘Balanced & Vegetable-Rich’), the OR was 0.47 (95% CI, 0.24, 0.91).

We observed that mothers who gave birth to girls and adhered to the ‘Balanced & Vegetable-Rich’ dietary pattern had significantly lower odds of their children developing AL compared to those who gave birth to boys. This difference was evident in both cluster and factor analyses. Cluster analysis demonstrated odds ratios (OR) of 0.40 (95% CI: 0.17, 0.94) for mothers following a ‘Balanced & Vegetable-Rich’ pattern, while mothers of boys had OR of 0.64 (95% CI: 0.2, 1.47). There is a significant interaction between the “Balanced & Vegetable-Rich” dietary pattern and the sex of infants in relation to the risk of AL (P for interaction 0.020). Similarly, factor analysis showed OR of 0.32 (95% CI: 0.11, 0.97) for mothers of girls adhering to a ‘Balanced & Vegetable-Rich’ pattern, in contrast to OR of 0.49 (95% CI: 0.19, 1.23) for mothers of boys, also showing a statistical significant pattern x sex interaction (0.037).

The ‘Moderate Meat & Cereals’ pattern was found to have an inverse association with the development of AL, with an OR of 0.28 (95% CI: 0.11, 0.97). This suggests that a higher intake of the ‘Moderate Meat & Cereals’ pattern is associated with reduced odds of AL. Notably, this association was significant overall (Table 3).

**Table 3:** Association between acute leukemia and dietary patterns.

	Total population		Girls		Boys	
Patterns	Cases/Controls	OR (95%CI)*	Cases/Controls	OR (95%CI)*	Cases/Controls	OR (95%CI)*
*Cluster*						
High Dairy & Cereals	41/65	Ref	24/25	Ref	17/40	Ref
Balanced & Vegetable-Rich	69/181	0.51 (0.29, 0.90)	34/74	0.40 (0.17, 0.94)	35/107	0.64 (0.27, 1.47)
P for trend		0.020		0.036		0.291
P for interaction pattern x sex		0.020				
*Factor*						
High Saturated Fats & Sugars						
T1	42/82	Ref.	27/20	Ref.	55/22	Ref.
T2	27/82	0.52 (0.27, 0.97)	42/18	0.56 (0.22, 1.42)	40/9	0.43 (0.18, 1.16)
T3	41/82	1.07 (0.57, 2.01)	30/20	1.12 (0.39, 3.22)	52/21	1.02 (0.45, 2.35)
P for trend		0.964				
P for interaction pattern x sex		0.558		0.927		0.901
Moderate Meat & Cereals						
T1	46/82	Ref.	26/34	Ref.	20/48	Ref.
T2	31/82	0.68 (0.38, 1.22)	18/31	0.56 (0.22, 1.42)	13/51	0.56 (0.24, 1.32)
T3	33/82	0.64 (0.34, 1.20)	14/34	0.28 (0.09, 0.82)	19/48	0.96 (0.41, 2.26)
P for trend		0.149				
P for interaction pattern x sex		0.044		0.021		0.843
Balanced & Vegetable-Rich						
T1	43/82	Ref.	22/33	Ref.	21/49	Ref.
T2	41/82	0.85 (0.48, 1.52)	24/34	0.89 (0.36, 2.18)	17/48	0.76 (0.33, 1.74)
T3	26/82	0.47 (0.24, 0.91)	12/32	0.32 (0.11, 0.97)	14/50	0.49 (0.19, 1.23)
P for trend		0.029				
P for interaction pattern x sex		0.037		0.051		0.129

*Adjusted by institution, residence, person/room ratio, maternal education, breastfeeding, maternal age at pregnancy, as well as maternal tobacco and alcohol consumption and supplement use of iron and vitamins during pregnancy.

## 4. Discussion

To our knowledge, this is the first study to examine childhood AL and dietary patterns in a sample of pregnant women in Mexico. Using two different approaches, we found two similar ‘Balanced & Vegetable-Rich.’ patterns, characterized by high consumption of foods included in the following groups: fruits, vegetables, allium, legumes, and low intake of refined sugars and cereals that were negatively associated with AL. These ‘Balanced & Vegetable-Rich.’ patterns were inversely and significantly associated with AL only among girls. In contrast, we found a positive but not significant relationship between AL and a pattern characterized by saturated fats and cereals.

There is no previous evidence on maternal dietary patterns and childhood leukemia, and the scarce information on food groups is inconclusive. According to a recent meta-analysis (8), a challenge in this area is to have information on comparable food groups across studies. In this context, for example, two studies included in a recent meta-analysis reported an inverse relationship between the consumption of fruits (OR: 0.81, 95% CI: 0.67–0.99), vegetables (OR: 0.51, 95% CI: 0.28, 0.94); and legumes (OR: 0.76, 95% CI: 0.62–0.94) with AL. Our results confirmed those associations in spite that there might be some different foods within the groups.

Several biological mechanisms have been implicated, as fruits and vegetables contain micronutrients that exert a protective action against leukemogenesis. Antioxidants, in particular vitamin A (retinoid acid), C (ascorbic acid) and E, as well as carotenoids are known to protect against oxidative damage of lipids, lipoproteins and DNA (4). Carotenoids have been shown to enhance DNA repair and have a positive effect on immune function, cell transformation and differentiation (4). Ascorbic acid can inhibit the *in vitro* proliferation of leukemic cells (4), while vitamin A plays a prominent role in the induction of terminal differentiation of lymphoid and myeloid blasts and in the inhibition of their clonogenic growth (4). In addition, direct and dose–response cytotoxic effects against leukemic cells have been suggested through selective regulation of cell cycle proteins for a variety of flavonoids present in most green leafy vegetables.

The consumption of allium vegetables, mainly garlic, onion, and leeks has not been studied regarding AL. Extensive experimental research has consistently shown the anticarcinogenic potential of allyl sulfides and flavonoids in relation to colon, gastric (particularly quercetin which is present abundantly in onion) and found that these compounds promote inhibition of mutagenesis, modulation of enzyme activities, inhibition of DNA adduct formation, free-radical scavenging, and effects on cell proliferation and tumor growth (17–22). However, epidemiological findings have not been conclusive. Previous meta-analyses have shown that high consumption of allium vegetables might be inversely associated with gastric and colorectal cancer (23, 24) Moreover a worldwide pooled analysis, reported an inverse association between allium vegetables intake and gastric cancer (25). In our sample we found an inverse relationship that warrant further attention since it could be possible that some of these mechanisms were the same for childhood AL.

Likewise, our results are consistent with the findings of studies that have suggested a positive association with sugar and refined grains, a study conducted in greek population, found that the odds of AL were higher with increased maternal intake of sugars and syrups (OR, 1.32; 95% CI, 1.05–1.67) (26). The potential mechanisms supporting the positive association between sugars and cancer, have already been discussed and include adiposity and insulin signaling pathway disruption, hormonal imbalances, inflammation, oxidative stress, DNA damage, and alteration of gene expression (27). Nevertheless, further research is needed to elucidate the relationship between AL and sugar intake.

The study of meat and processed meat consumption related to childhood cancer has been of interest since the 1990s. A more recent study, found that children who regularly ate cured meat (more than once a week) had a 74 percent greater chance of developing acute leukemia (28). Meat contains nitrosamines which have been classified as a type 1 carcinogen (29). Consumption of cured/smoked meat leads to the formation of carcinogenic N-nitroso compounds in the acidic stomach (30). Due to the high heterogeneity among the few epidemiological studies, a conclusion of the relationship between processed meats intake and AL is not currently stated. Our results do not suggest an association between meat and AL, consistently with the study by Ross et al. and in contrast to other studies. Since lack of statistical power may be and explanation for this, this question warrants further research.

The lower OR observed for mothers of girls following the ‘Balanced & Vegetable-Rich’ dietary pattern and the statistically significant pattern x sex interaction suggest that this dietary regimen may have a more pronounced protective effect against AL in female offspring. While the gender-specific differences in the impact of maternal healthy dietary patterns on leukemia risk are apparent in our findings, the exact biological mechanisms underlying these distinctions remain complex and not yet fully understood. Several biological factors could contribute to these gender-specific associations. One such factor is the influence of sex hormones, which play a vital role in the development and function of the immune system. Estrogens, for example, are known to have immunomodulatory effects and may affect the immune response against leukemic cells. Epigenetic modifications represent another plausible mechanism. Maternal diet during pregnancy can influence epigenetic changes in the developing fetus. These modifications can affect gene expression and cellular function, potentially contributing to variations in leukemia risk. Moreover, the immune system and its response to dietary patterns may differ between the sexes. It’s known that immune responses are inherently different in males and females due to differences in immune cell populations, immune regulatory pathways, and the expression of various immune-related genes (31).

A comprehensive review and meta-analysis, spanning 38 studies, published by Blanco-López (32) et al. in 2023, shed light on the role of maternal dietary factors in childhood acute leukemia. Notably, it highlighted a reduced risk of acute lymphoblastic leukemia with increased maternal fruit consumption, while heightened coffee intake was associated with an elevated risk. These findings are consistent with the results of our study. However, to craft effective population-level prevention strategies, further research, especially from high-quality cohort studies, is crucial for identifying causal factors in this complex landscape of childhood leukemia etiology.

Several limitations of this study should be considered to interpret our results. The extrapolation of our results is limited, since we had a low participation rate within the cases, and we did not have enough information from the children who did not participate to assess the representativeness of our sample. On the other hand, the maternal dietary information was not blinded to the case–control child status, however it is highly unlikely that the mothers reported, differentially between the groups, a pattern with a specific direction to be associated with AL. Nevertheless, since the collection of data on maternal nutrition during pregnancy took place around 3 years after birth, we cannot rule out the possibility of nondifferential measurement error, which translates into attenuation of the associations reported in this paper. As in all observational studies, confounding cannot be ruled out, therefore we adjusted for potentially confounding variables which were chosen after a careful analysis with the Daggity software (directed acyclic graph, Supplementary Figure 1). It’s important to acknowledge that we did not have access to data on certain well-established risk factors for AL, such as exposure to pesticides or infections, which could have served as potential confounding variables.

An additional limitation of our study is that we did not explore the potential influence of genetic factors in the Mexican population. Genetic epidemiology could provide valuable insights, as the genetic architecture of Mexican individuals may play a role in the observed associations. Future research could benefit from incorporating genetic analyses to comprehensively investigate the interplay between genetic predisposition and maternal dietary patterns in childhood leukemia risk.

Our findings suggest that a dietary pattern during pregnancy characterized by the high consumption of fruits, allium vegetables, other vegetables, and legumes and low in pastries and refined cereals may be associated with reduced odds of AL, mainly in girls. Further prospective studies with more detailed diet and biomarker assessments are necessary to confirm our findings, to elucidate potential mechanisms that explain the effect of the maternal dietary patterns according to infant sex. The results of this study emphasize the importance of promoting healthy maternal dietary patterns during pregnancy for the long-term health of the offspring.

## Data availability statement

The raw data supporting the conclusions of this article will be made available by the authors, without undue reservation.

## Ethics statement

The studies involving humans were approved by Comisión Nacional de Investigación Científica. The studies were conducted in accordance with the local legislation and institutional requirements. Written informed consent for participation in this study was provided by the participants’ legal guardians/next of kin.

## Author contributions

PM-A: Formal analysis, Methodology, Writing – original draft. ED-G: Formal analysis, Methodology, Writing – review & editing. MP-S: Data curation, Funding acquisition, Project administration, Writing – review & editing. LE-H: Data curation, Validation, Writing – review & editing. ED-A: Data curation, Validation, Writing – review & editing. JT-N: Data curation, Validation, Writing – review & editing. KS-L: Data curation, Validation, Writing – review & editing. RP-A: Data curation, Validation, Writing – review & editing. MV-A: Data curation, Validation, Writing – review & editing. RE-E: Data curation, Validation, Writing – review & editing. MM-M: Data curation, Validation, Writing – review & editing. AG-Á: Data curation, Validation, Writing – review & editing. LR-V: Data curation, Validation, Writing – review & editing. JD-H: Data curation, Validation, Writing – review & editing. JM-G: Data curation, Validation, Writing – review & editing. AC-E: Data curation, Validation, Writing – review & editing. ML-C: Data curation, Validation, Writing – review & editing. SM-S: Data curation, Validation, Writing – review & editing. JR-G: Data curation, Validation, Writing – review & editing. NH-P: Data curation, Validation, Writing – review & editing. JF-B: Data curation, Validation, Writing – review & editing. JP-G: Data curation, Validation, Writing – review & editing. MR-V: Data curation, Validation, Writing – review & editing. DT-V: Data curation, Validation, Writing – review & editing. JO-D: Data curation, Validation, Writing – review & editing. AM-R: Data curation, Validation, Writing – review & editing. LG-C: Data curation, Validation, Writing – review & editing. CA-H: Data curation, Validation, Writing – review & editing. JF-L: Data curation, Validation, Writing – review & editing. JN-E: Data curation, Validation, Writing – review & editing. MM-R: Data curation, Validation, Writing – review & editing. HR-V: Data curation, Validation, Writing – review & editing. DD-R: Data curation, Validation, Writing – review & editing. SJ-M: Data curation, Validation, Writing – review & editing. JM-A: Data curation, Funding acquisition, Investigation, Supervision, Writing – review & editing. LL-C: Conceptualization, Supervision, Writing – original draft, Writing – review & editing.
