# Supplementary material for: Maternal dietary patterns and acute leukemia in infants: results from a case control study in Mexico
Source: Front Nutr. 2023 Nov 13;10:1278255. doi: 10.3389/fnut.2023.1278255 (PMC10680405; doi:10.3389/fnut.2023.1278255)
Supplement: Supplementary file 1 [file Table_1.DOCX]

Supplementary Table 1. Foods and food groups used in the dietary pattern analysis.

| **Food groups and foods** | **Food items** |
| --- | --- |
| High fat dairy products | Milk, Oaxaca cheese, fresh cheese, manchego cheese |
| Dairy with added sugar | Ice cream, yogurt |
| Citrus fruits | Orange, orange juice, tangerine, tangerine juice, grapefruit, grapefruit juice |
| Other fruits | Banana, peach, apple, grapes, strawberries, melon, watermelon, mango, pear, cactus fruit, papaya, pineapple, plum, blackberry, mamey, zapote^a^ |
| Processed meats | Sausage, ham, chorizo, bacon |
| Red meat | Beef, pork, barbecue, carnitas, liver, cecina^b^ |
| Fish and shellfish | Tuna, shellfish, sardine, fish |
| Saturated fats | Pork rinds, sour cream, butter, mayonnaise, lard |
| Cruciferous vegetables | Broccoli, cauliflower, cabbage |
| Allium vegetables | Onion, garlic |
| Green leafy vegetables | Purslane, spinach, lettuce, parsley |
| Other vegetables | Zucchini, stewed tomato, raw tomato, chili, squash flower, chayote, nopal^c^ |
| Root vegetables | Potato, carrot, beetroot |
| Soybean products | Soy based drink (soy milk), tofu (soy chesse), textured soy protein (soy meat) |
| Legumes | Lentils, beans, broad beans, peas |
| Canned chili peppers | Canned chili peppers |
| Vegetable fats | Vegetable oil, margarine, corn oil, olive oil, soy oil |
| Cereals | Flour tortilla, white bread, rice, pasta, oatmeal, breakfast cereal, bolillo |
| Cereals high in fat and sugar | Cookies, cake, sweet bread |
| Soft & energy drinks | Soft drink, energy drink |
| Alcoholic drinks | Red wine, white wine, beer, liquors/spirits (rum, brandy, tequila) |
| Coffee and tea | Coffee, herbal tea, green tea, black tea |
| Atole | Atole^d^ |
| Avocado | Avocado |
| Dehydrated cranberries | Dehydrated cranberries |
| Eggs | Eggs |
| Poultry | Poultry |
| Corn | Corn |
| Soy sauce | Soy sauce |
| Corn tortilla | Corn tortilla |
|  |  |
| ^a^ local fruit, ^b^ local salted beef meat, ^c^ local cactus leaf, ^d^ local corn-based drink | |
