# Supplementary figures and images for: Maternal dietary patterns and acute leukemia in infants: results from a case control study in Mexico
Source: Front Nutr. 2023 Nov 13;10:1278255. doi: 10.3389/fnut.2023.1278255 (PMC10680405; doi:10.3389/fnut.2023.1278255)

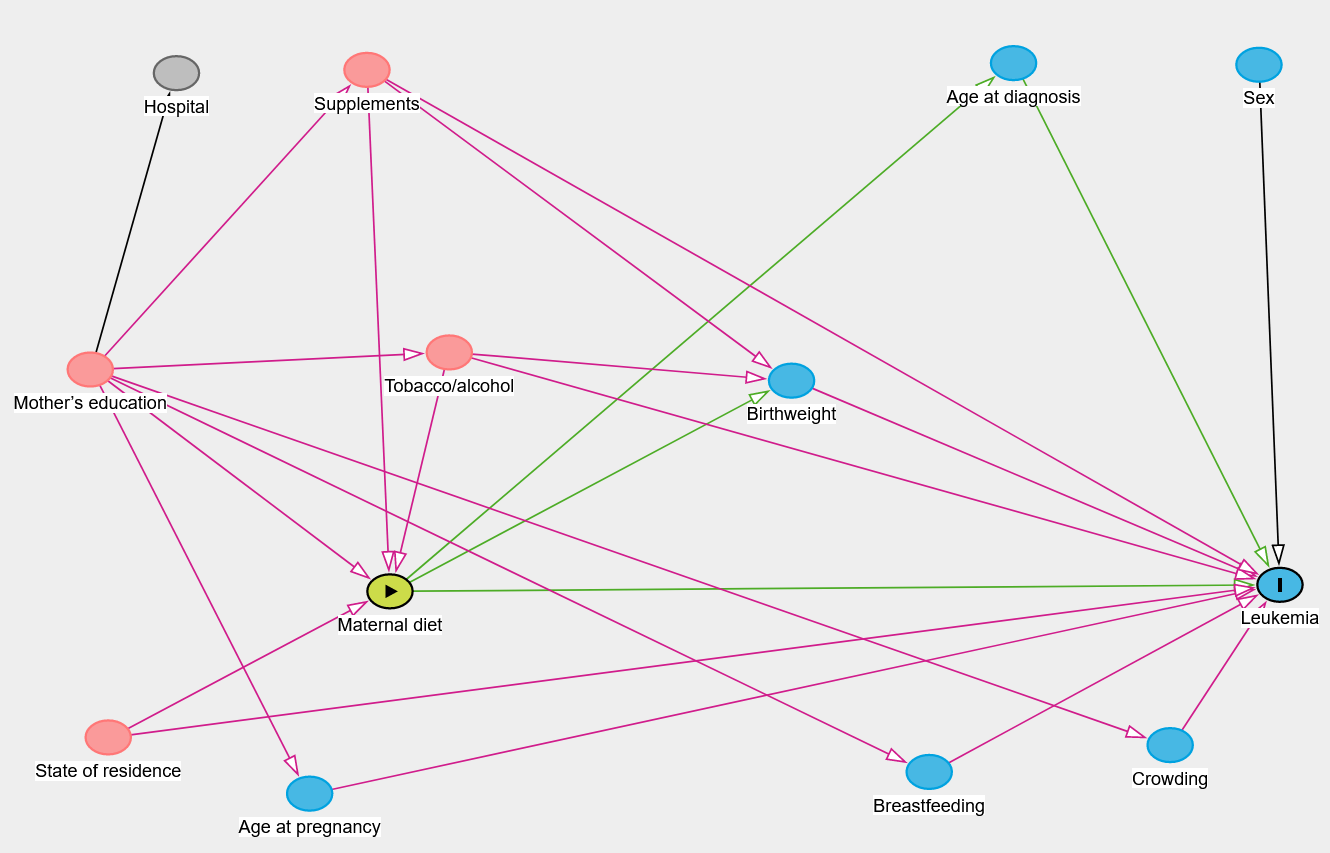

Supplement: Supplementary file 2 [file Image_1.PNG]
